# Adaptive fine-tuning based transfer learning for the identification of MGMT promoter methylation status

**DOI:** 10.1088/2057-1976/ad6573

**Published:** 2024-07-30

**Authors:** Erich Schmitz, Yunhui Guo, Jing Wang

**Affiliations:** 1 Advanced Imaging and Informatics for Radiation Therapy (AIRT) and Medical Artificial Intelligence and Automation (MAIA) Laboratory, Department of Radiation Oncology, University of Texas Southwestern Medical Center, Dallas, TX, United States of America; 2 Department of Computer Science, The University of Texas at Dallas, Richardson, TX, United States of America

**Keywords:** transfer learning, MGMT methylation status, SpotTune, deep learning, adaptive fine-tuning

## Abstract

*Background.* Glioblastoma Multiforme (GBM) is an aggressive form of malignant brain tumor with a generally poor prognosis. *O* ^6^-methylguanine-DNA methyltransferase (MGMT) promoter methylation has been shown to be a predictive bio-marker for resistance to treatment of GBM, but it is invasive and time-consuming to determine methylation status. There has been effort to predict the MGMT methylation status through analyzing MRI scans using machine learning, which only requires pre-operative scans that are already part of standard-of-care for GBM patients. *Purpose.* To improve the performance of conventional transfer learning in the identification of MGMT promoter methylation status, we developed a 3D SpotTune network with adaptive fine-tuning capability. Using the pretrained weights of MedicalNet with the SpotTune network, we compared its performance with a randomly initialized network for different combinations of MR modalities. *Methods.* Using a ResNet50 as the base network, three categories of networks are created: (1) A 3D SpotTune network to process volumetric MR images, (2) a network with randomly initialized weights, and (3) a network pre-trained on MedicalNet. These three networks are trained and evaluated using a public GBM dataset provided by the University of Pennsylvania. The MRI scans from 240 patients are used, with 11 different modalities corresponding to a set of perfusion, diffusion, and structural scans. The performance is evaluated using 5-fold cross validation with a hold-out testing dataset. *Results.* The SpotTune network showed better performance than the randomly initialized network. The best performing SpotTune model achieved an area under the Receiver Operating Characteristic curve (AUC), average precision of the precision-recall curve (AP), sensitivity, and specificity values of 0.6604, 0.6179, 0.6667, and 0.6061 respectively. *Conclusions.* SpotTune enables transfer learning to be adaptive to individual patients, resulting in improved performance in predicting MGMT promoter methylation status in GBM using equivalent MRI modalities as compared to a randomly initialized network.

## 1. Introduction

Glioblastoma Multiforme (GBM) is an aggressive malignant brain tumor characterized by a generally poor prognosis and low survival rate. While it has a relatively low incidence compared to other forms of cancers, it is the most common form of primary malignant brain tumors, accounting for 45.6% of them (Wirsching *et al* 2016). While there is one known risk factor for GBM, namely from ionizing radiation to the head, it does not account for all cases (Wirsching *et al* 2016). Coupling this with indeterminate onset symptoms, GBM is often diagnosed and treated late into its progression. Generally the treatment will involve surgical resection, radiation therapy, and concomitant/adjuvant temozolomide (TMZ), but even with these intensive treatments, the 2-year survival rate is only 26.5% (Stupp *et al* 2005). With such a low survival, especially with treatment, a major focus GBM research is to identify clinical bio-markers that can be used to predict how a patient will respond to treatment. One potential bio-marker is the methylation status of the *O* ^6^-methylguanine-DNA methyltransferase (MGMT) promoter which provides prognostic information on how a patient will respond to TMZ (Butler *et al* 2020).

One challenge associated with utilizing MGMT methylation as a clinical bio-marker is the complex process required to assess the methylation status. Current processes require biopsies or micro-surgical resection followed by a time-consuming molecular analysis, with no guarantees that there will be sufficient tissue available to perform a full analysis (Wirsching *et al* 2016). This lends to the need for a quick non-invasive process to determine methylation status. Several previous studies have explored potential in using deep learning to predict methylation status on MR imaging. Depending on the data used, there has been some success with using convolutional neural networks (CNNs) to predict the methylation status with a receiver operating characteristic (ROC) area under the curve (AUC) ranging from 0.58 − 0.91, but due to the size of many of these study’s datasets, ranging from 53 − 498 patients, it is difficult to assess their generalizability, but they indicate promise in using machine learning techniques to identify features correlated with MGMT methylation (Han *et al* 2018, Li *et al* 2018, Le *et al* 2020, Zlochower *et al* 2020, Adam Flanders 2021, Do *et al* 2022, Sakly *et al* 2023). There are two analyses that we have found that use the same dataset as this study. The first achieved an AUC of 0.598 while attempting to reduce their network’s parameter space through knowledge-based filtering of the MR images (Capuozzo *et al* 2022). Using a smaller number (182) of patients from the UPENN-GBM dataset, another study achieved an AUC of 0.81 using a radiomics based model that incorporates information from diffusion-tensor imaging (DTI) and dynamic susceptibility contrast (DSC) MR modalities (Tran Nguyen Tuan *et al* 2023).

In contrast to many applications involving natural images, the sample size of medical imaging is often limited. To address this challenge and train a CNN-based model effectively, transfer learning is widely adopted. Transfer learning leverages domain knowledge from a source task to enhance the performance of a target task, a practice commonly applied when the target task has a dataset that is limited in size (Guo *et al* 2018). Given that our classification task involves MR volumes, a related source task with valuable domain knowledge is medical image analysis/segmentation, which can be accessed through MedicalNet (Chen *et al* 2019), a collection of weights pre-trained on a multi-class segmentation task using 3D medical images. In this study, we employed transfer learning with adaptive fine-tuning to classify MGMT promoter status, which enables evaluation tailored to individual patients.

The concept of fine-tuning is closely related to transfer learning, as it involves the optimization of the transferred network weights. In the context of this study, fine-tuning specifically refers to parameters that are allowed to adjust during training, while freezing refers to parameters that remain constant throughout training. In conventional transfer learning tasks, the choice of which parameters to fine-tune is manually determined by trial-and-error, with the most common practice being to fine-tune parameters within the last layer/s of the network. The choice of which parameters in a network to fine-tune involves a delicate balance between enabling the network to learn more about the target data by increasing the amount of fine-tuned parameters (thus risking overfitting), and preserving domain knowledge from the source task through frozen parameters (potentially not effectively learning the target dataset). To automate the choice of which parameters to fine-tune, we turn to adaptive fine tuning, eliminating the need for trial-and-error in finding the best combination of fine-tuned parameters. Due to the small size of many medical image datasets, coupled with the variability in patient characteristics, it can be difficult to extract meaningful image features in a network where all the parameters are fine-tuned. With the addition of transfer learning, image features found in the training of larger datasets are extracted, while the adaptive fine-tuning introduces a way to account for variability in image characteristics by fine-tuning on a patient-by-patient basis, allowing the network to extract the most applicable features for each image.

In this project, we developed a SpotTune-based adaptive fine-tuning approach for methylation status prediction in GBM using MRI, where the previously developed SpotTune algorithm dynamically navigates through the fine-tuned and frozen layers within a Residual Network (Guo *et al* 2018). We specify a Residual Network, as it has been shown to be resilient to the exchange of residual blocks since each block acts as a shallow classifier (Guo *et al* 2018). In a SpotTune network the dynamic navigation is implemented through the exchange of residual blocks. Specific to SpotTune, the dynamic routing is determined on a per image basis, which is ideal for medical imaging due to patient variability. As part of this study, the SpotTune framework, originally developed for 2D imaging classification tasks, was extended to handle full 3D MR volumes. A key consideration in our approach is that SpotTune use the Residual Network, which matches the network that the transfer weights are sourced from, and additionally has a straightforward implementation. With regards to its performance, in its initial conception, SpotTune was compared against other fine-tuning models in the Visual Decathlon challenge, and achieved the highest score based on its performance in 10 different image datasets (Guo *et al* 2018). To the best of our knowledge, this study represents the first use of transfer learning with adaptive fine-tuning in MGMT promoter methylation status, while other studies have only considered traditional transfer learning (Sakly *et al* 2023).

## 2. Methods and materials

### 2.1. Dataset

The University of Pennsylvania glioblastoma (UPENN-GBM) cohort is a collection of 630 patients that were diagnosed with glioblastoma, and is freely available to use via The Cancer Imaging Archive (Clark *et al* 2013, Bakas *et al* 2021, 2022). The dataset includes magnetic resonance scans with perfusion and diffusion derivatives, computational and manually derived annotations of tumor regions, radiomic features of the tumor regions, and clinical and molecular information (Bakas *et al* 2022). Included in the molecular information is the mutational status of IDH and the MGMT promoter methylation status.

Of the 630 patients, 611 have preoperative scans comprising of four structural MRI scans: T1, post-contrast gadolinium enhanced T1 (T1-GD), T2-weighted, and T2 fluid attenuated inversion recovery (FLAIR). A subset of these patients also have diffusion tensor imaging (DTI) and dynamic susceptibility contrast (DSC) scans. The DTI scans have 4 derivative volumes: tensor’s trace, axial diffusivity, readial diffusivity and fractional anisotropy (Bakas *et al* 2022). The DSC scans have 3 derivative volumes: peak height, percentage signal recovery, and an automated proxy to the relative cerebral blood volumes (Bakas *et al* 2022). There are 291 patients that have the MGMT promoter methylation status available, of these there are 262 who have the corresponding pre-operative scans with 151 not methylated and 111 methylated. The provided images used were already converted from the DICOM format into the Neuroimaging Informatics Technology Initiative (NIfTI) format, following the processing protocol of the Brain Tumor Segmentation (BraTS) challenge (Bakas *et al* 2022). This preprocessing included de-identification, de-facing, re-orientation of images to the left-posterior superior coordinate system, registration and resampling to an isotropic resolution of 1 *mm* ^2^ based on the SRI common anatomical atlas, and an N4 bias field correction (Bakas *et al* 2022). Additionally segmentation masks are provided for 3 tumor subregions, the enhancing tumor, necrotic tumor core and the edematous tissue. These masks were automatically generated with some manual refinement where needed.

From the full cohort, 240 patients and their imaging were selected for this study. These correspond to the patients that have a pre-operative base scan and the MGMT methylation status available, minus 22 that were dropped due to how the training datasets between different modalities were split. For the structural imaging modalities the 240 patients are used, while for the DTI and DSC volumes, there are 208 and 189 patients respectively. Of the patients with a DSC perfusion scan, 108 are not methylated and 81 are methylated, and for those with DTI scans 127 are not methylated and 91 are methylated.

In table 1 we summarized some of the basic characteristics of the 240 selected patients. In the selection there are 1.7 times as many males as females. However, the distribution of methylated and unmethylated samples is similar between the genders, with ratios of 0.7 for males and 0.8 for females. The age of the patients ranges from 18 to 70+ years, with a majority of them falling into the 50-69 years range. For the older age groups there are similar numbers of methylated compared to unmethylated patients, while for the younger age groups (18-29 & 30-49 years) the distribution favors the unmethylated class, with 83% and 73% of the patients in these age groups, respectively, falling under the unmethylated class.

**Table 1.** The patient characteristics of the 240 patients selected for analysis. The percentages for the characteristics are relative to the total number, 240. The percentages for the methylated and unmethylated are relative to the particular characteristic.

Patient characteristics				
		Number (%)	Methylated (%)	Unmethylated (%)
Gender				
	Male	150 (62.5%)	62 (41.3%)	88 (58.7%)
	Female	90 (37.5%)	40 (44.4%)	50 (55.6%)
Age (years)				
	18-29	6 (2.5%)	1 (16.7%)	5 (83.3%)
	30-49	23 (9.6%)	6 (26.1%)	17 (73.9%)
	50-69	146 (60.8%)	64 (43.8%)	82 (56.2%)
	70+	65 (27.2%)	31 (47.7%)	34 (52.3%)

### 2.2. Data preparation

The images provided in the UPENN-GBM dataset were pre-processed following the protocol set forth by the International Brain Tumor Segmentation (BraTS) challenge (Bakas *et al* 2021). This pre-processing made the image dimensions and voxel sizes uniform across the different scanners, acquisition protocols, and modalities. These images were manually inspected at different steps, and were corrected or realigned when necessary. Using the pre-processed NIfTI files provided in the UPENN-GBM dataset, we applied a further pre-processing before the images were used for training and testing. The images and masks were read in using the SimpleITK software package (Lowekamp *et al* 2013), and converted into numpy arrays for ease of use. The image masks were applied, and the size of the images were reduced by cropping following the largest tumor among the patients, and down-scaling the images by a factor of 2 to give a 2 *mm* spacing. The cropping was done to both reduce the size of the image and to only encompass the size of the largest tumors among the available images. This reduced the dimensions of the volumes respectively from 155 × 240 × 240 → 140 × 172 × 164 → 70 × 86 × 82. For the final image size, the last dimension was padded to have a dimension of 70 × 86 × 86 to provide squared dimensions for the slices to ease the accounting for dimensions in the DNN. Lastly the images had a min-max scaling applied separately for each patient, with a resulting image range of [0, 1] in order to preserve the shape of the distribution of voxel values while maintaining the relative distance between them.

The dataset was split into a training and testing set at a 70:30 ratio, chosen to increase the size of the testing dataset. For training and validation, we initially looked at using a train/validation/test splitting where the validation was set as a quarter of the training set. In order to increase the robustness of the model, we switched to 5-fold cross validation (CV) within the training dataset, after the 70:30 split between the training and testing sets, where four of the folds are used for training and the fifth for validation. This came out to having a training set of 183, 151, and 132 patients for the structural, DTI, and DSC modalities respectively and a common testing set of 57 patients. The datasets were split such that the different MR modalities would share patients in the training and testing sets, i.e., the patients that are in the testing set for the DSC derivatives will be the same set of patients contained in the testing set of the structural modalities. So that the results are directly comparable between modalities, only the patients common to the DSC modalities were retained in the testing dataset for the structural and DTI modalities. After the training set was split into its 5 folds, each set of 4 folds used for training were augmented in four ways including flipping the images, randomly rotating the images between −180 and 180 degrees, adding Gaussian noise, and applying an elastic deformation (van Tulder 2022). Different combinations and numbers of augments were assessed, and based on validation performance, we chose one of each of the four augments, where our initial choice of which specific augments to apply came from what is commonly used in literature Chlap *et al* (2021). To account for the class imbalance of the dataset, the augmented training folds were randomly pruned until the ratio of the MGMT classes were equal. The class imbalance was kept in the validation and testing datasets.

### 2.3. Model

The 3D SpotTune network developed for this study is an amalgamation of three residual networks (ResNets) that allows for a dynamic routing through sets of fine-tuned and frozen pre-trained residual blocks. As SpotTune was initially produced to run on 2D images, it was further developed to run on 3D images. The bulk of the conversion to a 3D network involved changing out the 2D convolutional layers with their 3D counterparts. Additionally, the padding of the layers needed to be adjusted for the shape of the 3D inputs, due to the differences in the dimensions. The rest of the adjustments for the 3D network were done during the network training, involving the optimization of hyper-parameters specific to a SpotTune network, detailed in section 2.4. The optimization of these hyper-parameters ended up being the most challenging part of implementing the 3D network, as the 3D SpotTune network has 40.5 million more trainable parameters than its 2D counterpart, increasing from 19.5 to 60 million.

The SpotTune network consists of three separate ResNets, one agent and two base networks. The base neural network used is a ResNet50 (He *et al* 2015), which consists of 16 residual blocks adding up to 50 layers in total. Two of these networks make up the main network, where one ResNet50 contains a set of frozen residual blocks, while the other contains fine-tuned blocks. The agent network is a ResNet10 and is used to make the policy that the main network follows in how to route the data through the frozen and fine-tuned blocks. With 16 residual blocks for the ResNet50, the agent network has 16 classes as output, with 2 categories each corresponding to whether a block is to be frozen or fine-tuned. The routing is determined following (1), describing the *ℓ*-th residual block, where *x* is the input image, *F*_*ℓ*_ is the *ℓ*-th block from the frozen ResNet50, ${\hat{F}}_{{\ell }}$ is the *ℓ*-th block from the fine-tuned ResNet50 and a duplicate of *F*_*ℓ*_, and *I*_*ℓ*_(*x*) is a binary variable taken from the policy output by the agent network (Guo *et al* 2018).

\begin{eqnarray*}{x}_{{\ell }}={I}_{{\ell }}(x){\hat{F}}_{{\ell }}({x}_{{\ell }-1})+(1-{I}_{{\ell }}(x)){F}_{{\ell }}({x}_{{\ell }-1})+{x}_{{\ell }-1}\end{eqnarray*}

**Figure 1.**
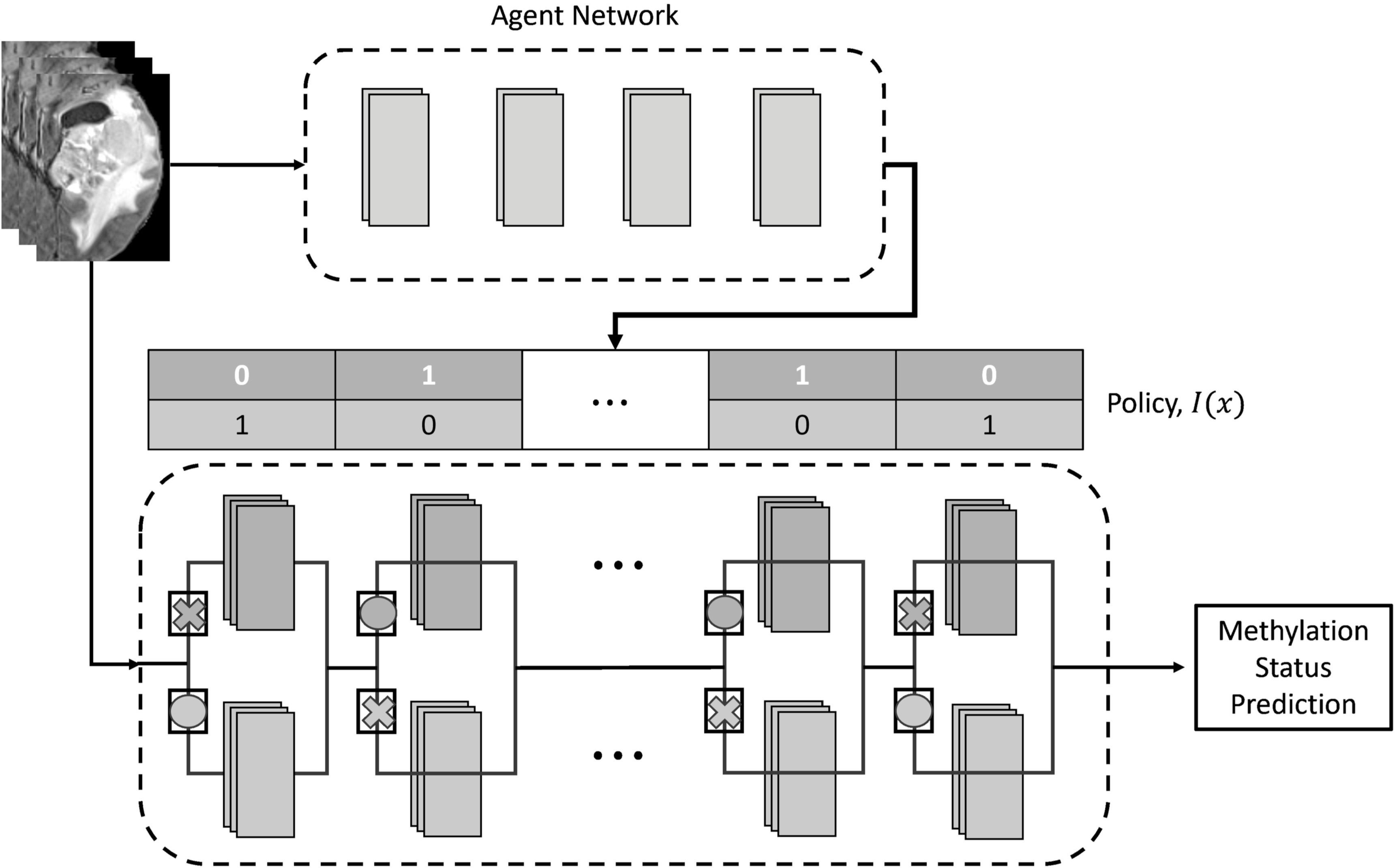
A diagram illustrating the SpotTune Network. The Agent network produces the policy used by the main network. The main network takes this policy, shown as a vector of binary values, and routes inputs through the residual blocks of the main network, with the routing illustrated using X’s and O’s.

The policy output by the agent network is a collection of binary variables that is discrete and non differentiable, so it is difficult to calculate the gradients of the agent network based on the predictions of the full network. In order to allow back-propagation of the gradient through the full network a Gumbel Softmax sampling approach is used (Guo *et al* 2018). In the forward pass of the network a Gumbel distribution built using the output by the agent network is used to produce the policy for the main network following (2), where *α*_*i*_ corresponds to the output of the agent network, *G*_*i*_ is the standard Gumbel distribution, and *X* corresponds to a discrete sampling of the policy, *I*_*l*_(*x*) (Guo *et al* 2018). The purpose of this discrete sampling is to provide the switches between the frozen and tine-tuned blocks of the main network, giving a path for the data to flow through. In the backward pass, instead of the discrete values needed by the main network, what is instead needed is a continuous distribution for which the gradients can be calculated. To do this the Gumbel Softmax distribution is used as a continuous relaxation of (2), and is given in (3), where *τ* is the temperature parameter that controls how discrete the resulting distribution is and is an important parameter in our tuning process (Guo *et al* 2018). By sampling from this distribution, the originally discrete policy is made continuous and differentiable, allowing the back propagation of the policy through the agent network. Figure 1 illustrates the SpotTune network and how it would route inputs through the main network based on a policy given by the agent network.

\begin{eqnarray*}X=\arg \,\max [\mathrm{log}{\alpha }_{i}+{G}_{i}]\end{eqnarray*}

\begin{eqnarray*}{Y}_{i}=\displaystyle \frac{{\exp }((\mathrm{log}{\alpha }_{i}+{G}_{i})/\tau )}{{\sum }_{j=1}2{\exp }((\mathrm{log}{\alpha }_{j}+{G}_{j})/\tau )}\end{eqnarray*}

When the agent network creates a routing policy, it does so for each image, *x*, provided as input. This means that each image gets a unique policy, *I*(*x*), that determines how blocks should be swapped in the main network. In the forward pass of training, the agent network will give a discrete policy for each input, that the main network will then follow, swapping between the frozen and fine-tuned residual blocks as designated. In the backward pass the gradient from the main network will be back propagated through the agent network allowing it to refine the policy. The agent network is then jointly trained with the main network to determine the optimal fine-tuning strategy and thus maximize the performance metric (Guo *et al* 2018). Additionally, with the proposed adaptive fine-tuning, depending on the input images, each patient will have a unique route through the fine-tuned blocks of the pre-trained model, which can lead to a more personalized prediction.

In addition to the SpotTune network, a regular ResNet50 is used for comparison purposes. The ResNet50 is initialized with two different sets of weights, corresponding to random weight initialization following a uniform distribution and the transferred MedicalNet weights as used in the SpotTune network. The ResNet50 with transferred weights is initialized so that only the classification layer is fine-tuned, keeping all other layers frozen.

### 2.4. Network training and evaluation

The networks were trained using the pytorch framework (Paszke *et al* 2019) with a maximum of 90 epochs, a batch size of 12, and a starting learning rate of 1e-5 for the main network and 1e-4 for the agent network. The learning rate followed a decrease-on-plateau based scheduler with a patience of 20 epochs and a multiplicative factor of 0.1. Additionally, the temperature, *τ*, of the Gumbel Softmax distribution was set to 100. The objective function used in the network was binary cross-entropy loss, with the Adam algorithm used for optimization. The inputs to the network are single channel volumes corresponding to each available modality/derivative for a total of 11 modalities: 4 structural, 4 DTI, and 3 DSC. Before deciding on training with a single channel, we also looked at multi-channel inputs ranging from 3 up to 11 channels, with each channel corresponding to a modality/derivative. In order to implement multiple channels the MedicalNet weights had to be adjusted since they assume single channel inputs. To accomplish this adjustment, we followed a weight inflation method that duplicates weights along a desired axis, and averages them over the number of duplications (Zhang *et al* 2022). For example, for a 3-channel input, the weights of the first convolutional layer are duplicated 3 times along the channel axis, with those weights averaged over the 3 channels. The network was trained using 5-fold cross-validation and evaluated using the average precision (AP), area under the receiver operating characteristics curve (AUC), sensitivity (SEN), and specificity (SPE) metrics from the torchmetrics package (Detlefsen *et al* 2022). The AP and AUC are the main metrics used in place of accuracy due to the data imbalance, since higher accuracies did not guarantee a high true positive rate. The AUC gave us an overall performance for each model, since it is ideal in showing performance for all classification thresholds in a binary prediction. We evaluated SEN and SPE to get a better of idea of how balanced they were, since we found that choosing models based on accuracy and AUC often gave highly unbalanced results, with a low SEN and high SPE. The best models to be used for the hold-out testing dataset were determined by evaluating the performance of the validation folds using an evaluation criterion, *M*, based on the sensitivity and specificity, and given in (4) (Chen *et al* 2021).

\begin{eqnarray*}M=0.6\times ({SEN}\geqslant 0.5)+0.4\times ({SPE}\geqslant 0.5)\end{eqnarray*}

As part of the training process the hyper-parameters were tuned using a random grid search to get an initial parameter set. With this set as basis, further independent scans were performed on a selection of hyper-parameters. These independent scans included the number of epochs with a range of 30 − 200, learning rate with a range of 0.1 − 1*e* − 8, and Gumbel Softmax temperature, with a range of 1*e* − 4 − 1*e*4. The ranges for these scans were chosen to encompass their likely values. For the learning rate we chose a maximum value close to 1 and minimum close to zero, with the actual values being arbitrary. We gave the learning rate special consideration as the network was sensitive to overfitting, as a smaller rate reduced the over-fitting but prevented the network from learning as much of the target dataset, while a larger learning rate allowed more learning on the target dataset, but also caused it to move away from the source weights and increasing the overfitting. The range of epochs was chosen for a minimum where the trainings started to stabilize up to a maximum beyond where the trainings tended to plateau. The temperature range was chosen to encompass multiple orders of magnitude in a attempt to characterize how changes in the order will affect the overall training. The metric used for validation was initially a combination of ACC and AUC, but discovered that this metric selected models with a severely unbalanced sensitivity and specificity. To rectify this, we changed to a metric that prefers a balanced sensitivity and specificity, as seen in (4), following what was done in another study (Chen *et al* 2021). As mentioned previously, a major concern of training with 3D inputs is the sensitivity to overfitting due to the large number of parameters, so as part of the hyper-parameter scan we looked at the degree of overfitting that was occurring on the training dataset.

The models chosen for evaluation on the testing dataset were combined into various sets of ensembles based on their imaging modalities and network used. Due to the random sampling inherent to the Gumbel Softmax distribution, each model created using the SpotTune network was evaluated 100 times using a random seed of 42. These 100 samples were combined to form the chosen model for each of the folds in order to better encompass the distribution of probabilities possible from the random sampling. The chosen models for the CV folds were combined by taking the mean of the output probabilities to get a single model for each imaging modality, and then the models for derivatives of each modality were combined in the same way to get overall models. The models presented in the results fall under 3 categories: those using the SpotTune network, those using randomly initialized weights in a regular ResNet50 and those that use the transferred weights in a regular ResNet50. For each of these categories, the overall models that were evaluated are labeled as DSC, DTI, Structural, and then combinations of the three. The DSC and DTI models are ensembles of their derivative volumes, and the Structural model is an ensemble of the four structural modalities T2, FLAIR, T1, and T1GD. The additional modality combinations correspond to DSC and DTI (DSC+DTI), DSC and Structural (DSC+Struct), DTI and Structural (DTI+Struct), and the three together (DSC+DTI+Struct) in order to cover the possible permutations of the three groupings. While no single modality was excluded from the evaluation, the groupings we chose may not necessarily correspond to the highest possible performance combinations. We combine the similar modalities together for three categories (structural, DSC, DTI) to simplify the number of models, and to focus on comparing the different architectures. This may end up biasing the performance, as what we end up with is a combination of each modality grouping, where individual modalities may adversely affect the overall performance.

In addition to the deep learning models from this study, we compared our results with another model that achieved superior performance (AUC = 0.88 on a private dataset) in predicting MGMT promoter methylation status using a set of 6 radiomic features in a random forest classifier (Li *et al* 2018). Rather than directly comparing the published results with ours, we followed their workflow and attempted to reproduce and train their model on our dataset, due to the difference in datasets. Following the published model, we calculated the 6 radiomic features that were used: (1) Skewness from the T1 core, (2) energy from the T1 edema invasion, (3) GLCM contrast from the FLAIR necrotic core, (4) GLSZM gray level variance from the T1GD enhanced area, (5) GLSZM low gray level zone emphasis from the T2 edema invasion, and (6) NGTDM busyness from the T2 core. Each of these features came from different modalities and masks, where the core refers to the combination of the enhanced area and the necrotic core (Li *et al* 2018). The features were used as input to a random forest classifier and trained with the same dataset splitting as this study. The optimal hyperparameters of the random forest were set as following: a number of estimators of 10, 000, a maximum tree depth of 5, and a minimum number of samples at a leaf node of 10.

## 3. Results

We compare ensemble models for three different network categories. The main comparisons are between models using the same inputs, only considering cross modality comparisons for the top models for each network category. Tables 2, 3, and 4 give the results for the SpotTune, random weight initialization, and transferred weight initialization respectively. Included in the tables are the AUC of ROC curves and the Average Precision of precision-recall curves (AP), with the AP given to show how well the models classify the positive, i.e. methylated, samples. Additionally the sensitivity and specificity are included to better show any imbalance in how the models classify the positive and negative samples.

**Table 2.** The ROC AUC, average precision, sensitivity and specificity evaluated on the hold out testing dataset using model ensembles created with the SpotTune network. The highest values for the ROC AUC and AP are given in bold text.

SpotTune results				
Model	AUC	AP	SEN	SPE
DSC	0.6124	0.5842	0.5000	0.6061
DTI	**0.6654**	0.6008	0.7500	0.6364
Struct	0.5707	0.5346	0.5833	0.4848
DSC+DTI	0.6604	**0.6179**	0.6667	0.6061
DSC+Struct	0.6073	0.5962	0.5417	0.5455
DTI+Struct	0.6376	0.5289	0.5833	0.6364
DSC+DTI+Struct	0.6389	0.5779	0.6667	0.5758

**Table 3.** The ROC AUC, average precision, sensitivity and specificity evaluated on the hold out testing dataset using model ensembles created with a ResNet50 initialized with random weights. The highest values for the ROC AUC and AP are given in bold text.

Randomly initialized weights results				
Model	AUC	AP	SEN	SPE
DSC	0.5051	0.4872	0.3750	0.5758
DTI	0.6023	0.5159	0.3750	0.6364
Struct	**0.6061**	**0.5334**	0.4167	0.6061
DSC+DTI	0.5707	0.5127	0.3333	0.6364
DSC+Struct	0.5669	0.5271	0.3750	0.6667
DTI+Struct	0.5997	0.5187	0.4167	0.6667
DSC+DTI+Struct	0.5783	0.5018	0.4583	0.6364

**Table 4.** The ROC AUC, average precision, sensitivity and specificity evaluated on the hold out testing dataset using model ensembles created with a ResNet50 initialized with transferred MedicalNet weights, while only fine-tuning the classification layer. The highest values for the ROC AUC and AP are given in bold text.

Transferred weights results				
Model	AUC	AP	SEN	SPE
DSC	0.5303	0.4421	0.5000	0.6970
DTI	**0.5947**	**0.5445**	0.5833	0.4848
Struct	0.4722	0.4250	0.5417	0.4242
DSC+DTI	0.5808	0.4992	0.5417	0.5455
DSC+Struct	0.5051	0.4296	0.5000	0.5455
DTI+Struct	0.5732	0.4869	0.6250	0.6061
DSC+DTI+Struct	0.5682	0.4788	0.5000	0.6061

**Figure 2.**
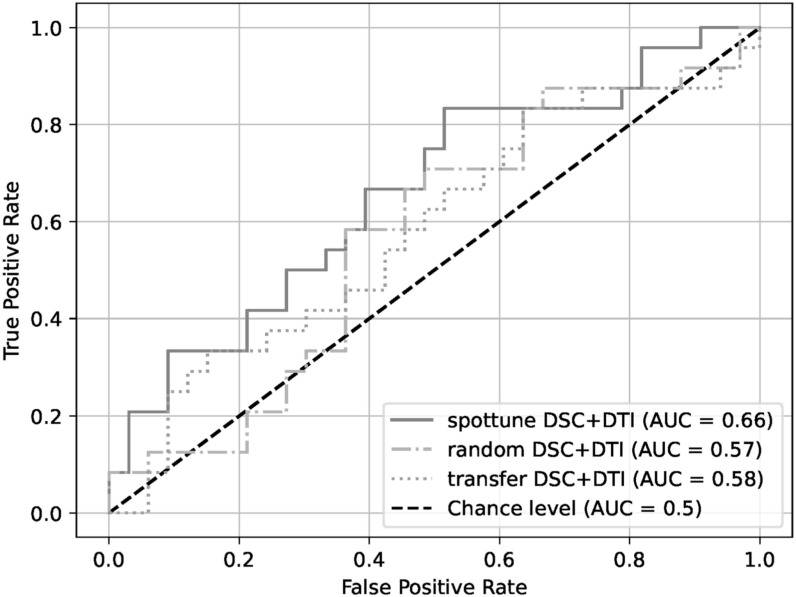
ROC curves for the DSC+DTI model ensembles for the three network categories.

**Figure 3.**
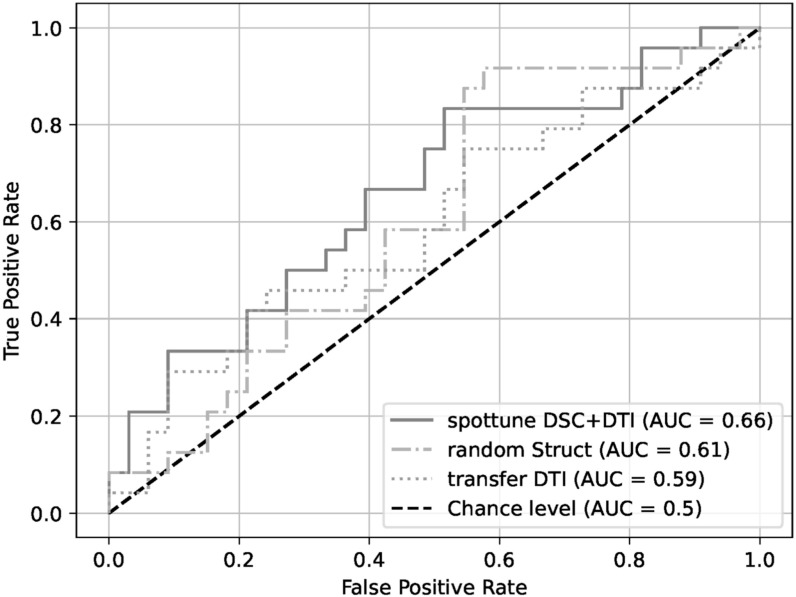
ROC curves for the best overall model ensembles for the three network categories.

**Figure 4.**
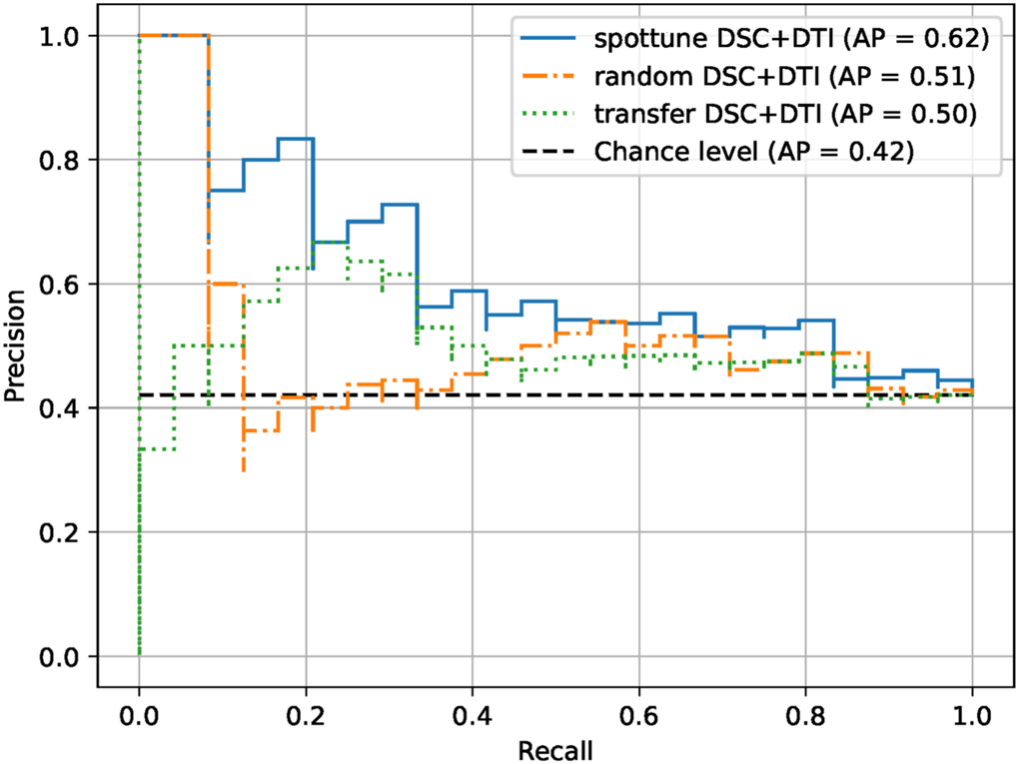
Precision-recall curves for the DSC+DTI model ensembles for the three network categories.

**Figure 5.**
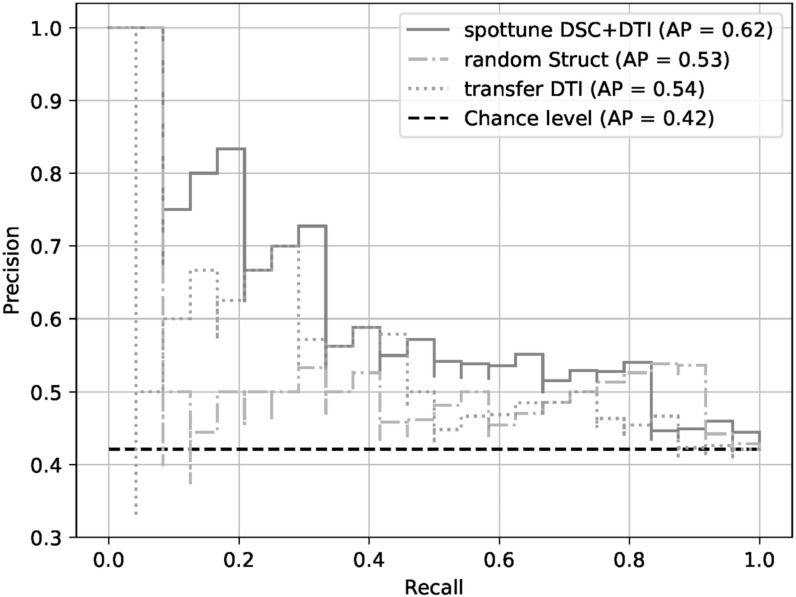
Precision-recall curves for the best overall model ensembles for the three network categories.

With the exception of the model using the structural modalities on their own, the SpotTune network outperforms the randomly initialized network for similar inputs. The highest overall performance is given by the DSC+DTI SpotTune model with a ROC AUC of 0.6604, and an AP of 0.6179. These results outperform the corresponding DSC+DTI model using the ResNet50 with randomly initialized weights, which had a ROC AUC of 0.5707 and an AP of 0.5334. The same DSC+DTI model using transferred weights with a ResNet50 showed a slightly better performance when only looking at the ROC AUC of 0.5808, but the picture changes when also considering the AP of 0.4992, showing the the non-adaptive transfer learning is not sufficient on its own. ROC Curves of the DSC+DTI models for each of the network categories can be found in figure 2, and curves for the overall best models for the three categories is found in figure 3. Additionally the precision-recall curves are given for the same ensembles in figures 4 and 5.

In addition to the improved overall performance of the SpotTune models for similar inputs, there is a noteworthy observation with regards to the sensitivity and specificity. The SpotTune models have a better balance and higher sensitivity than the randomly initialized models in all of the ensembles. The sensitivity for the randomly initialized model tends to be low, with a *SEN*/*SPE* ratio as low as 0.5, with a maximum sensitivity between the models of 0.4583, compared to the maximum in the SpotTune models of 0.7500. Applying traditional transfer learning as shown in table 4 improves the sensitivity/specificity balance over the randomly initialized weights and the addition of the SpotTune network further improves it.

For the comparison radiomics model on the dataset used in this study, the random forest classifier with 6 chosen radiomic features achieved a ROC AUC of 0.5764, an AP 0.4886, a sensitivity of 0.2174 and a specificity of 0.9355. Since this comparison model only made use of the structural modalities we can compare it to the SpotTune model that used the structural modalities as input. The ROC AUC of the two models are similar at 0.5707 and 0.5764, but the SpotTune gave a better AP of 0.5346 compared to the 0.4886 of the comparison radiomics model. For the best overall SpotTune model, both the ROC AUC and AP showed an improved performance. Additionally the SpotTune models had a better balance between the sensitivity and specificity, compared to the high imbalance for the comparison radiomics model.

The significance, *p* < 0.05, of the results was checked by performing a Friedman chi-square test followed by a post-hoc Nemenyi test should the Friedman test prove significant. The p-values for the two sets tests are given in table 5, and compare the models that share the same input between the SpotTune, non-adaptive transfer learning, and randomly initialized networks. From the Friedman test, four sets of models were significant with *p* < 0.05: The DSC+DTI, DSC+Struct, DTI+Struct and DSC+DTI+Struct. Of these four sets of models, four specific comparisons showed a significant difference in the results following the Nemenyi test. Three of the comparisons corresponded to the SpotTune and randomly initialized networks, with the last between the non-adaptive transfer learning and randomly initialized networks. It is noteworthy that each SpotTune network that showed significance involved the DTI modalities as part of their input, but only when coupled with other modalities. If we loosen the significance to *p* < 0.1 the best overall models then pass the Friedman test, with each set of comparisons showing significance using the Nemenyi test.

**Table 5.** p-values calculated from the Friedman chisquare and nemenyi tests for comparing the spottune, randomly initialized and non-adaptive transfer learning networks. Comparisons are between models that used the same inputs, and then one for the best overall model from each network. The best overall models compared are the DSC+DTI, Struct, and DTI for the SpotTune, randomly initialized and non-adaptive transfer learning networks, respectively. Comparisons that show significance, *p* < 0.05, are shown in bold.

Friedman chi-square & Nemenyi p-values			
Model	Friedman p-value	Networks compared	Nemenyi p-value
DSC	0.108	SpotTune, Random	0.177
		Random, Transfer	0.147
		SpotTune, Transfer	0.9

DTI	0.128	SpotTune, Random	0.121
		Random, Transfer	0.339
		SpotTune, Transfer	0.822

Struct	0.0970	SpotTune, Random	0.0982
		Random, Transfer	0.249
		SpotTune, Transfer	0.876

DSC+DTI	**0.0131**	SpotTune, Random	**0.0103**
		Random, Transfer	0.147
		SpotTune, Transfer	0.554

DSC+Struct	**0.0222**	SpotTune, Random	0.0503
		Random, Transfer	**0.0395**
		SpotTune, Transfer	0.9

DTI+Struct	**0.00606**	SpotTune, Random	**0.00567**
		Random, Transfer	0.0635
		SpotTune, Transfer	0.661

DSC+DTI+Struct	**0.0180**	SpotTune, Random	**0.0181**
		Random, Transfer	0.0982
		SpotTune, Transfer	0.768

Best Overall	0.0636	SpotTune, Random	0.0181
		Random, Transfer	0.0982
		SpotTune, Transfer	0.0768

## 4. Discussion and conclusion

In this study we examine how transfer learning with adaptive fine-tuning has the potential to improve the prediction of MGMT methylation status over the use of randomly initialized weights in similar networks. Using the SpotTune network, we showed improved results compared to randomly initialized counterparts for various model ensembles. It is well known that the volume of data in a machine learning experiment can greatly affect performance, and dataset size is an especially important concern with relation to medical imaging (Chen *et al* 2019). Through the use of transfer learning, the lower level information that is gained from a large dataset can be applied to a smaller dataset with varying degrees of fine-tuning. With transfer learning specifically, a concern in medical imaging is the availability of weights trained on a sufficiently large dataset that also contains necessary domain knowledge. While it is possible to use non-medial domain pre-trained weights, there is no guarantee that the low level features that can be extracted are applicable to the medical images, especially in the 3D domain where 2D pre-trained weights are missing important spatial information that associates the slices of the 3D image. Additionally pre-trained weights from datasets of videos and 3D objects do not translate as well to 3D medical images due to their structure. In essence, the dataset characteristics inform on what type of pre-trained weights would have the largest impact on the training performance. This concern is partially abrogated through the MedicalNet weights which are trained on 3D segmentation tasks using MRI and CT scans from eight public medical datasets. While the dataset is not as large as the ImageNet dataset, which is more commonly used in transfer learning tasks, it contains 3D domain knowledge more specific to the target task. As the weights are produced from a multi-institutional dataset the method of adaptive fine-tuning could generalize to other tasks involving medical imaging. Closely tied to this is the choice of architecture where, specifically for adaptive fine-tuning, the resilience of the network to be able to swap out layers can greatly impact performance. With the ResNet, the residual blocks act as distinct networks daisy-chained together, so swapping blocks does not negatively affect the training. The addition of adaptive fine-tuning to the tranfer learning works to remove a layer of complexity in the training, namely optimization of which layers to fine-tune and freeze. This difference between traditional fine-tuning and adaptive fine-tuning is seen in the improvement of results in table 2 over table 4, not including the reduction in the usage of computational resources since there was no need to run dozens of trainings to optimize the selection of fine-tuned layers.

We compared our results to multiple studies, two of which used the same dataset (Capuozzo *et al* 2022, Tran Nguyen Tuan *et al* 2023), and another in which we attempt to reproduce their results on the dataset used in this study (Li *et al* 2018). Capuozzo *et al* (2022) reported a ROC AUC of 0.5980, a sensitivity of 0.4535 and a specificity of 0.7403. Their strategy involved using 2D and 3D image and knowledge based filtering in place of a segmentation mask to define a region of interest. Since the study only looked at the structural modalities, they were able to cross validate their results with an external dataset, the BraTS 2021 challenge dataset (Menze *et al* 2015, Bakas *et al* 2017, Adam Flanders 2021, Baid *et al* 2021), though their results showed poor generalization between models trained on one and tested on the other. This is also shown in the other study using the UPENN-GBM dataset (Tran Nguyen Tuan *et al* 2023), where they found the model based on a genetic algorithm random forest (Bakas *et al* 2017), performed worse on the UPENN-GBM dataset compared to Bakas *et al* (2017)'s private dataset. By using features from the DTI and DSC modalities, Tran Nguyen Tuan *et al* (2023) achieved a ROC AUC of 0.81, a sensitivity of 0.78 and a specificity of 0.84 using 182 patients from the UPENN-GBM dataset. This particular study ended up with similar findings to our own, but with a better performance. Both studies found that the DSC and DTI modalities had better discriminating power compared to the use of only structural modalities, which could explain some of the differences between our results, and the results of Capuozzo *et al* (2022). There are two main differences between our study and the radiomics based study from Tran Nguyen Tuan *et al* (2023), the overall patient selection and the chosen testing cohort. Tran Nguyen Tuan *et al* (2023) selects 182 patients, compared to our 240, where the difference arises from the number of patients that have a DSC modality in addition to the structural or DTI. Additionally, differences in performance may be related to the differences in the testing cohort between the two studies, with both having small testing cohorts where the differences in selection can change the evaluation.

The model we attempted to reproduce from Li *et al* (2018) originally had a ROC AUC of 0.88, a sensitivity of 0.70 and a specificity of 0.86, while our model only produced a ROC AUC of 0.5764, an AP of 0.4886, a sensitivity of 0.2174 and a specificity of 0.9355. This is a stark difference to what was originally reported, and could be due to bias in the datasets, since they come from different institutions. The difference could also be attributed to our reproduction as the original study only mentioned the names of the features and what classification models were used, but there was not further information on how the images were processed before calculating the features and there were not any comments on what hyper-parameters were used, necessitating our own parameter tuning, and producing radiomics features using images that were not necessarily pre-processed in the same way. From these results, along with the results from Capuozzo *et al* (2022) who used an external dataset, even though our results do not necessarily perform as well as the studies mentioned in the introduction section, (Han *et al* 2018, Li *et al* 2018, Le *et al* 2020, Zlochower *et al* 2020, Do *et al* 2022, Sakly *et al* 2023), it is important to note that the differences in datasets make direct comparisons difficult. This denotes a need for an increase in reproducibility and data availability to determine the actual relative performance between these different models.

The data availability problem was partially solved through the BraTS 2021 challenge, which provided a large multi-institutional dataset of over 600 training cases for the identification of MGMT methylation with a separate external testing dataset (Menze *et al* 2015, Bakas *et al* 2017, Adam Flanders 2021, Baid *et al* 2021). This challenge, though, did not give optimistic results, with the best model only having a ROC AUC of 0.62, denoting a lack of underlying features that can help with the identification of MGMT methylation. One problem, though, is that the provided images do not have manually refined segmentation masks, and the dataset only contains the structural modalities which we have found to have a worse performance in MGMT methylation identification. Other studies besides ours, including the study that uses the same UPENN-GBM dataset (Tran Nguyen Tuan *et al* 2023), have found that DSC and DTI modalities apart from the normal structural ones show correlations between features of these modalities and the occurrence of MGMT and IDH-1 mutations (Moon *et al* 2012, Ryoo *et al* 2013, Choi *et al* 2021, Ozturk *et al* 2021, Tran Nguyen Tuan *et al* 2023).

With regards to optimizing the fine-tuned layers in traditional transfer learning, it may be noticed that there is one outlier in table 4, where the Struct model shows a performance less than 0.5 for the AUC. One potential reason for this is that the lower level features selected by the transferred weights do not correlate as well with the structural modalities, since the classification layer is the only one that is fine-tuned. Due to this, there were a number of training folds that were not able to produce a model that passes the selection criteria, forcing the training to use the model from the last epoch. For the Struct model, there were 5 training folds that were not able to produce models that passed the selection criteria, whereas this number was only 3 and 1 for the DSC and DTI models, respectively. To address this a second round of training was done for the structural modalities using the ResNet50 with transferred weights that relaxed the number of frozen layers, allowing the last two residual blocks, in addition to the classification layer, to be fine-tuned. The resulting model gave a more reasonable performance with an AUC of 0.5341, an AP of 0.5134, a SEN of 0.5417, and a SPE of 0.4848. These results support the idea that the strictness of the fine-tuning posed challenges for the Struct model, and that the transferred weights used were not as useful for these modalities. This also points to adaptive fine-tuning as a useful alternative as it will determine the best order of frozen and fine-tuning based on a machine learning architecture, rather than by hand. Should the transferred weights not be as useful for extracting meaningful features for a given input, the adaptive fine-tuning will allow the network to rely solely on the fine-tuned parameters. Other alternatives would amount to trying different sets of pre-trained weights and network architectures, apart from the MedicalNet weights and ResNet, to see if the inputs will generalize better with those. Determining if there is a better combination of network architecture and pre-trained weights is a possible avenue of future research.

In addition to the use of adaptive fine-tuning for improved results, the choice of imaging modalities also plays an important role in performance. In this study, each ensemble model was made by combining models trained on separate imaging modalities made available in the UPENN-GBM dataset. Important decisions included whether these different modalities should be included as separate channels in a single training, and which combination of models, if any, would yield optimal results. When considering the number of channels, it was found that keeping the trainings to a single channel was optimal, especially with regards to the MedicalNet weights, which assumes a single channel. This can be seen in a separate training done using the 3 DSC derivatives as 3 channels in a single training, resulting in an AUC of 0.5758, compared to the AUC of 0.6124 given in table 2 for the DSC model, which is an ensemble of the 3 DSC derivative models. For the 3-channel case the weights were generated using a technique known as weight inflation (Zhang *et al* 2022), where the single channel weights were duplicated and normalized for 3 channels. An interesting outcome related to the choice of imaging modality was the improved performance of models that used the diffusion and perfusion modalities over the more common structural modalities. While we give an overall performance for each modality grouping, one limitation of this study is that we do not fully explore the possible single modality combinations. The reasoning for this was to focus on the overall modality groupings rather than individual modalities, as it is easy to get bogged down in the possible permutations of 11 total individual modalities/derivatives. Future studies could explore this aspect to determine which single modality combinations provide the best performance and from this, which types of features correlate better with the MGMT methylation status, especially when looking at combinations of the DSC and DTI derivatives. One possible avenue to explore this is to do a ranked scoring of the individual modalities, much how one would rank the features in a radiomics based analysis, and then make an ensemble based on the highest ranked modalities

The main limitation of this work is the size of the dataset, with only 262 patients having the MGMT methylation status labeled. With the small sample size and the large heterogeneity present in GBM cases, the results of this study may not be generalized well to other datasets, as the training samples can affect the stability of the model. One such example of the lack of stability is the need for taking multiple samplings of a model to get an average prediction from a sparse Gumbel distribution. While transfer learning was used to counteract the small dataset size, it still proved an issue. To increase the size of the dataset, it would be possible to move from prediction of MGMT methylation status to one of treatment response in a future study. Such a course would have the potential of doubling the size of the dataset with respect to this study, since the overall dataset has 630 patients. For the MGMT classification task, some ways to account for the small dataset size would be to further increase the number of augmentations or to change the augmentation strategy. In this study, we did a single iteration of 4 different types of augmentations, but another strategy is to do multiple iterations of augmentation on the base dataset, such as applying a random rotation to all of the images of a dataset for a predefined number of angles.

With regards to the dataset itself, there are some limitations corresponding to it being a public dataset from a separate institution. As a public dataset it is difficult to supplement with any extra information since we do not have access to the original patient data, and additionally it is difficult to cross check the accuracy of the supplied data, and instead must be taken on good faith. A potential bias that arises from the public dataset corresponds to the labels provided for the methylation status. The methylation status is determined from a well accepted method by studying CpG sites for methylation, but there does not seem to be a set standard for how much methylation should constitute a methylated label, where there is a possibility that what would be considered methylated could be slightly different depending on the dataset and institution. For example, the UPENN-GBM dataset uses a an average percentage of 4 CpG sites to determine methylation. For an average methylation greater than 10% the patient is given a methylated label, while an average methylation less than 4.5% is negative, with additional cutoffs for a low positive and indeterminate label (Bakas *et al* 2022). Another study uses a cutoff value 8% to determine a positive methylation status, with no mention of averaging among multiple CpG sites (Bady *et al* 2012, Le *et al* 2020). These small differences in labeling could produce a bias in different datasets, affecting their comparability. Additionally, since methylation is a percentage rather than strictly a yes or no, a binary label is not necessarily ideal.

Other biases in the dataset generally correspond to the present imbalances, mostly with regards to the demographics and the MGMT methylation status label. For the MGMT status, we try to account for this through artificially balancing the classes before training through sample augmentation. This is done by augmenting more of the smaller class of samples than the larger class, until the two have the same numbers. A second approach to this is applying a class weight that places more emphasis on the smaller class, making them more important in the training. Two other imbalances that could bias the results are gender and age, where there are more examples of males than females, and a larger population of patients over the age of 50. For both of these cases the most common way to account for these would be to concatenate the features with the extracted image features in the classification layers. There is also the option of treating both age and gender as categorical features, and augmenting the dataset further to balance out these classes, similar to what we did for the MGMT methylation classes.

In the future, more can be done to study the correlations between the perfusion and diffusion modalities and MGMT methylation status, especially with regards to the base images, where current studies mostly look at radiomics features produced from these modalities. Further studies can be done to predict overall survival using the imaging features, perhaps incorporating pre-trained features related to MGMT methylation status prediction should there be further improvements.

## Data Availability

The data that support the findings of this study are openly available at the following URL/DOI: https://doi.org/10.7937/TCIA.709X-DN49.

## Conflict of interest

The authors have no relevant conflicts of interest to disclose.

## Code availability

The code used for this study is publicly available on GitHub at https://github.com/LabAIRT/SpotTune_MGMT_prediction.
